# Improved Optical and Electrochromic Properties of NiO_x_ Films by Low-Temperature Spin-Coating Method Based on NiO_x_ Nanoparticles

**DOI:** 10.3390/ma11050760

**Published:** 2018-05-09

**Authors:** Xiaohong Xie, Changkang Gao, Xiang Du, Gangyi Zhu, Weiguang Xie, Pengyi Liu, Zhenfang Tang

**Affiliations:** 1 Siyuan Laboratory, Guangzhou Key Laboratory of Vacuum Coating Technologies and New Energy Materials, Guangdong Provincial Engineering Technology Research Center of Vacuum Coating Technologies and New Energy Materials, Department of Physics, Jinan University, Guangzhou 510632, China; xiexiaohong0521@163.com (X.X.); gao625276@gmail.com (C.G.); du110488@sina.com (X.D.); tlpy@jnu.edu.cn (P.L.); 2 Guangdong Tecsun Vacuum Technology Engineering Co. Ltd., Zhaoqing 526060, China; 13197753659@163.com

**Keywords:** NiO_x_ nanoparticles, NiO_x_ films, optical and electrochromic properties, spin-coating method

## Abstract

Solution approaches to NiO_x_ films for electrochromic applications are problematic due to the need of an additional high-temperature annealing treatment step in inert gas. In this study, nanostructured NiO_x_ powder with grain size of about 10.1 nm was synthesized for fabrication of NiO_x_ films for electrochromic application. Non-toxic dispersants of isopropanol and deionized water were used and the whole process was carried out in air. The effects of the number of spin-coating layers, annealing temperature, and the volume ratios of isopropanol to deionized water were systematically investigated. Large transmittance change of 62.3% at 550 nm, high coloration efficiency (42.8 cm^2^/C), rapid switching time (coloring time is 4 s, bleaching time is 3 s), and good stability were achieved in the optimized NiO_x_ film. The optimized process only required a low processing temperature of 150 °C in air with spin-coating three times and 1:2 volume ratio of isopropanol to deionized water. Finally, good cycle durability of up to 2000 cycles without obvious degradation was demonstrated by cyclic voltammetry tests in a LiClO_4_/propylene carbonate electrolyte. This study provides a simple and effective approach for fabrication of NiO_x_ films at low temperature in air, which is attractive for further commercialization of electrochromic devices.

## 1. Introduction

According to statistics, building energy consumption accounts for 23–50% of total energy consumption [1]. Heat losses from windows and doors account for 20–30% of the whole building energy consumption [2]. Therefore, building energy conservation has become a hot topic. Electrochromism refers to reversible, persistent and visible change in transmittance or reflectance that is associated with an electrochemically induced oxidation-reduction reaction [3,4,5]. Electrochromic (EC) smart windows are a good choice to reduce the heat losses from windows due to their low energy consumption, lack of pollution and automatic adjustment of their optical properties [6]. 

Metal oxide material is a big family of inorganic EC materials that have been intensively studied [7,8,9,10,11,12,13]. They have excellent radiation resistances, high chemical stability, strong adhesions to substrates, and light weight over large areas [14]. Among all inorganic EC materials, NiO is one of the most important anodic coloring material due to its high optical modulation, fast responding time between coloring and bleaching processes, excellent durability, long-lasting memory, abundant raw materials and low-cost [10]. Complementary EC devices with NiO-based films as counter electrode layers have been extensively studied [11,12,13]. Chemical solution methods such as sol-gel methods [15,16,17,18,19], hydrothermal method [20,21,22] and chemical bath deposition (CBD) [23,24] have been widely used to fabricate NiO-based films for EC devices. Several ways are generally investigated to improve the optical and EC properties of NiO films. The first is to optimize the processing parameters such as thickness [12], annealing temperature [22], etc. Second is to improve the electrical properties by doping metal ions such as Al^3+^ [18], B^3+^ [25], Li^+^ [26], Co^+^ [23] in NiO films. Third is to make NiO-based films with multilayers [19,24,27,28]. Although the optimized optical and EC properties of NiO-based films are now acceptable in application, there is still one problem that hampers the reduction of fabrication cost and commercialization. High temperature annealing above 300 °C is required to improve the optical and EC properties of NiO-based films. In addition, protective gas or vacuum conditions are normally required in the fabrication process. Solving this problem becomes more important because of the increasing requirement of flexible EC devices, which must be fabricated on a substrate that cannot withstand temperature higher than 200 °C.

In this study, we showed the fabrication of NiO_x_ films with superior optical and EC properties that required low-temperature annealing at 150 °C in air by the spin-coating method. We optimize the EC properties of NiO_x_ films as a function of the number of spin-coating layers, annealing temperature, and the volume ratios of isopropanol (IPA) to deionized water (DI water) in the IPA-DI water-NiO_x_ suspensions (NiO_x_ inks). NiO_x_ film with large transmittance change, high coloration efficiency, rapid switching time and good cyclic stability is obtained. We also discussed the microscopic differences between NiO_x_ films to obtain a clear understanding of the differences in EC properties. 

## 2. Materials and Methods

### 2.1. Fabrication of Samples

**Figure 1:**
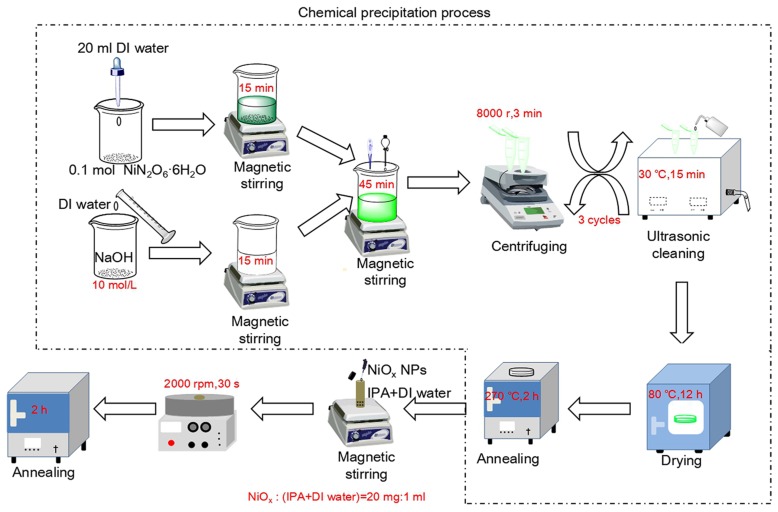
Preparation flowchart of NiO_x_ NPs and NiO_x_ films.

There are two methods to fabricate NiO_x_ films with different numbers of layers. One is to spin multiple layers and anneal the films at the end. In this study, we choose this method to reduce the processing complexity. The other is to anneal the films whenever each layer is coated. Although this method is complex, it is reported to further improve the film quality, which will be studied later [29]. Figure 1 shows the fabrication flowchart of NiO_x_ NPs and NiO_x_ films. Firstly, we fabricated high-quality non-stoichiometric NiO_x_ NPs similar to the facile chemical precipitation method of Fei Jiang and co-workers’ work [30]. 0.1 mol nickel nitrate hexahydrate (NiN_2_O_6_·6H_2_O, AR, 98%) was added into 20 mL DI water. After stirring, it formed a light green solution. Thenceforth, NaOH (AR, 98%) solution with a concentration of 10 mol/L was slowly added into the obtained solution until pH = 10. At this time, the green Ni(OH)_2_ colloidal precipitation was observed. The mixture was centrifuged and cleaned with an ultrasonic bath in DI water and circulated 3 times in turn. Then, the Ni(OH)_2_ precipitation was collected and dried at 80 °C for 12 h. Finally, this dried green product was annealed at 270 °C for 2 h in air to decompose into ultrafine dark-black NiO_x_ NPs. The NiO_x_ NPs can be stored for a long time for multiple uses. Reactions (1) and (2) illustrate the chemical reactions in this procedure of non-stoichiometric NiO_x_ NPs [30]:

(1) $$\mathrm{Ni}{\left(\mathrm{NO}\right)}_{3}+\mathrm{NaOH}\to \mathrm{Ni}{\left(\mathrm{OH}\right)}_{2}↓+\mathrm{Na}{\left({\mathrm{NO}}_{3}\right)}_{2}$$

(2) $$\mathrm{Ni}{\left(\mathrm{OH}\right)}_{2}\overset{270\;{}^{\circ}C}{\to }{\mathrm{NiO}}_{x}+H_{2}O$$

Secondly, we prepared NiO_x_ inks. 20 mg of the above NiO_x_ NPs were uniformly dispersed in 1 mL IPA-DI water mixture using magnetic stirring.

Thirdly, we fabricated NiO_x_ samples by the spin-coating method. Conductive indium tin oxide (ITO, 10 Ω/sq) coated glasses were cleaned with acetone, ethanol, and DI water in an ultrasonic bath for 15 min in sequence before use. The above NiO_x_ inks were spin-coated onto substrates with different numbers of coating layers (2000 rpm for 30 s each time). Finally, the NiO_x_ samples were annealed for 2 h in air for the evaporation of the organic solvent. 

### 2.2. Characterization

The morphology and structure of NiO_x_ films and NiO_x_ NPs were characterized by a field emission scanning electron microscope (FE-SEM, Zeiss Ultra 55, Carl Zeiss, Jena, Germany) and a XRD diffractometer (XRD, MiniFlex600, Cu K_α_ radiation, Rigaku, Tokyo, Japan). Electrochemical measurements of NiO_x_ electrodes were performed by employing a three-electrode electrochemical workstation (Versa STAT 3, AMETEK, Oak Ridge, TN, USA) and carried out in a three-electrode system in 1 M KOH (AR, 90%) electrolyte: the as-prepared sample was used as working electrode, an Ag/AgCl electrode and a platinum wire were used as reference and counter electrodes, respectively. Before each electrochemical test, each sample was first circulated 12 times by applying square-wave-type voltages (±1.7 V, 60 s per cycle) until the responses become stabilized. The transmission spectra of NiO_x_ films in fully colored and fully bleached states was measured over the wavelength range from 340 to 900 nm with a UV-vis spectrophotometer (Model UV-2550, Shimadzu, Tokyo, Japan). The transmittance of ITO-glasses in the 1 M KOH electrolyte was considered to be 100% transmittance and was used as the baseline.

## 3. Results

### 3.1. Microstructure Characteristics of the NiO_x_ NPs

**Figure 2:**
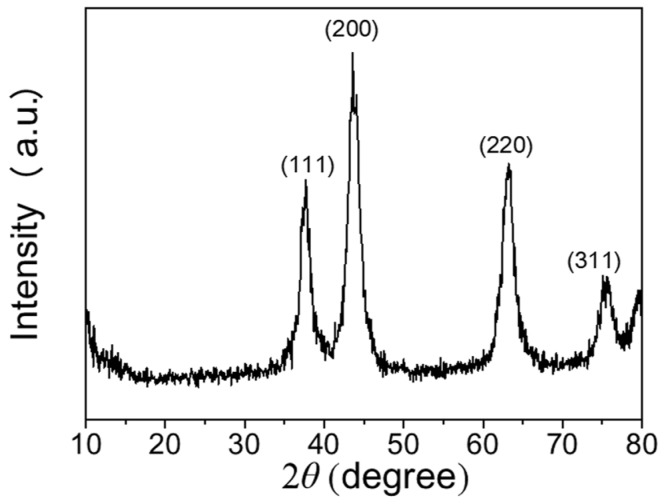
XRD pattern of NiO_x_ NPs fabricated by chemical precipitation method.

Figure 2 shows the XRD patterns of NiO_x_ NPs. Four prominent characteristic diffraction peaks of NiO_x_ cubic structure appears at 37.7°, 43.6°, 63.2° and 75.8°, belonging to the (111), (200), (220) and (311) planes (JCDPS No. 47-1049), the full width half maximum (FWHW) of the diffraction peaks are 0.805°, 0.878°, 0.978°, and 0.901°, respectively. No other peaks were observed. The crystallite size of the NiO_x_ NPs can be estimated from the four XRD diffraction peaks by Debye-Scherrer formula [31]:

(3) $$D=\frac{0.89\lambda }{B\cos\theta }$$

where *D* is the size of crystallite, *B* is the FWHW. *θ* is the Bragg angle (degree) and *λ* (0.154056 nm) is the wavelength of the X-ray. The average NiO_x_ crystallite size is estimated to be is 10.1 nm. The small grain size is favorable for ions to shorten diffusion pathway and increase the switching speed of NiO_x_ films and increase the utilization efficiency of active materials. Such characteristics are favorable for a fast EC reaction [32,33].

### 3.2. Optical and EC Properties of NiO_x_ Films

The coloring processes of NiO_x_ electrodes can be attributed to the following oxidation reactions [28]:

(4) $$\mathrm{NiO}+OH^{-}↔\mathrm{NiOOH}+e^{-}$$

Or

(5) $$\mathrm{NiO}+H_{2}O↔\mathrm{NiOOH}+H^{+}+e^{-}$$

The bleaching processes of the NiO_x_ electrodes can be attributed to the following reduction reaction:

(6) $$\mathrm{NiOOH}+H^{+}+e^{-}↔\mathrm{Ni}{\left(\mathrm{OH}\right)}_{2}$$

Or

(7) $$\mathrm{NiOOH}+H_{2}O+e^{-}↔\mathrm{Ni}{\left(\mathrm{OH}\right)}_{2}+{\mathrm{OH}}^{-}$$

**Figure 3:**
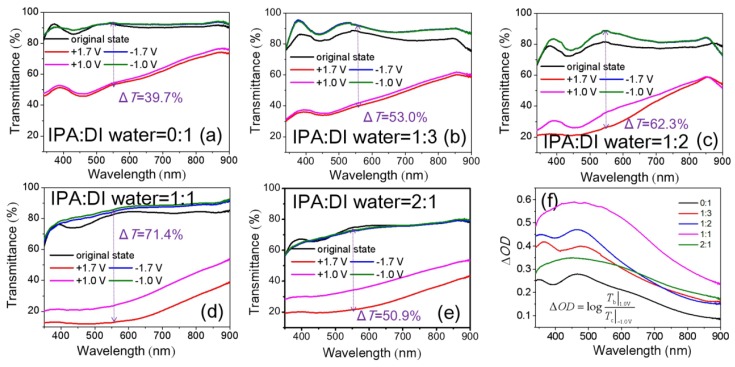
Optical transmittance spectra at as-deposited, colored and bleached states of NiO_x_ films as a function of the number of spin-coating layers: (**a**) spin-coating once; (**b**) spin-coating three times; (**c**) spin-coating five times; (**d**) spin-coating seven times (the increases of voltages has little effect on the *T*_b_, so the 2 bleached curves overlap together); (**e**) Δ*OD*; (**f**) Switching curve of the NiO_x_ film by spin-coating three times. The NiO_x_ films are annealed at 100 °C and the volume ratio in NiO_x_ inks is IPA:DI water = 1:3.

Δ*T* and switching time are the most important criteria indexes to evaluate optical and EC properties of materials. Δ*T* (*T*_b_ − *T*_c_) is the change of transmittance between the bleached states (*T*_b_) and the colored states (*T*_c_). The switching time is defined as the time required for a system to reach 90% of its full Δ*T*. Guofa Cai et al. [12] reported the optical and EC properties of inkjet-printed NiO films as a function of the number of printed layers. Sahu et al. [34] also reported the optical and EC properties of e-beam evaporated NiO films were affected with different thicknesses. Similarly, we studied the transmittance spectra of NiO_x_ films at colored and bleached states as a function of the number of spin-coating layers. The results are shown in Figure 3a–d. Colored states of the NiO_x_ films is obtained through applying positive voltages and bleached states by negative voltages. As the positive voltages increase from +1.0 V to +1.7 V, the transmittance of the colored states obviously decreases. However, the changes of the transmittance of the bleached states is not obvious when the negative voltage increases from −1.0 V to −1.7 V. The Δ*T* exhibits the best value of 50.5% at 550 nm varying from 93.0% to 42.5% between the bleached state (−1.7 V) and the colored state (+1.7 V). For the NiO_x_ films after spin-coating three times, the Δ*T* gradually becomes lower. The change in optical density (Δ*OD*) is defined as [5]

(8) $$\Delta OD\left(\lambda \right)=\log\frac{T_{b}\left(\lambda \right)}{T_{c}\left(\lambda \right)}$$

Δ*OD* represents the contrast between colored states and bleached states and it is presented in Figure 3e. Under the first spin-coating, the NiO_x_ NPs partially cover the surface of ITO-glass, the Δ*OD* is small. Under the third spin-coatings, the surface is gradually filled, forming a complete layer, which shows the best Δ*OD*. Further increase in numbers of spin-coatings increases the thicknesses of the NiO_x_ films. Because NiO and NiOH are semiconductors with extra-high resistances, the superfluous NiO_x_ NPs do not contact with electroconductive ITO layer and are less active in electrochemical processes. Figure 3f shows the changes in transmittance at 550 nm of the NiO_x_ film after spin-coating three times for applied square-wave-type voltages (±1.0 V, 60 s per cycle). It is calculated that the switching time is 3 s for the coloring process and 2 s for the bleaching process. The fast switching speed of the NiO_x_ film is attributed to the short diffusion pathways in the NiO_x_ NPs, which facilitates charge, transport [32,33]. 

**Figure 4:**
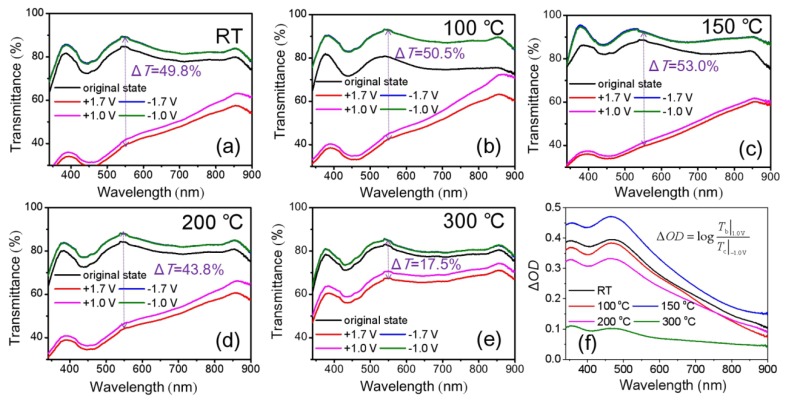
Optical transmittance spectra at as-deposited, colored and bleached states of the NiO_x_ films as a function of annealing temperature: (**a**) RT; (**b**) 100 °C; (**c**) 150 °C; (**d**) 200 °C; (**e**) 300 °C (The increases of voltage has little effect on the *T*_b_, so the 2 bleached curves overlap together) and (**f**) Δ*OD*. The NiO_x_ films are spin-coated three times and the volume ratio in NiO_x_ inks is IPA:DI water = 1:3.

**Figure 5:**
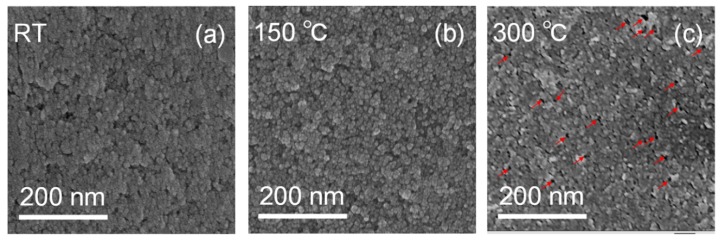
SEM images of NiO_x_ films after (**a**) RT; (**b**) 150 °C and (**c**) 300 °C annealing. The NiO_x_ films are spin-coated three times and the volume ratio in NiO_x_ inks is IPA:DI water = 1:3. The red arrows indicate the voids.

Chen et al. [22] and Gamze Atak et al. [35] reported that annealing temperature is also an important parameter affecting the optical and EC properties of NiO-based films. Similarly, we studied the transmittance spectra of NiO_x_ films by spin-coating three times as a function of the annealing temperature for 2 h in air after spin-coating. Figure 4a–e shows the transmittance spectra of NiO_x_ films at colored and bleached states as a function of annealing temperature. As annealing temperature is 100–150 °C, the transmittance of the bleached states can be improved to over 90.0%. The Δ*T* of NiO_x_ films annealed at room temperature (RT), 100 °C, 150 °C, 200 °C and 300 °C are 49.8%, 50.5%, 53.0%, 43.8% and 17.5% respectively between bleached states (−1.7 V) and colored states (+1.7 V). The Δ*OD* of the NiO_x_ film annealed at 150 °C is the highest Δ*OD* compared to the others (Figure 4f). These differences of annealing effects can be explained by the changes of SEM morphology in Figure 5. If the NiO_x_ film was not annealed, it can be seen that the NiO_x_ NPs was not obvious because of the presence of residuary dispersants and organic binders (Figure 5a). Uniform and obvious NiO_x_ NPs were observed after annealing at 150 °C as shown in Figure 5b. This was because the evaporation of the residuary dispersants and the decomposition of the organic binder led to an active electrochemical reaction of the NiO_x_ NPs with the KOH electrolyte after annealing at the appropriate temperature [12]. At higher annealing temperature, the nanoparticles aggregated, and some voids formed between these NiO_x_ NPs at the surface in Figure 5c. The voids would cause leakage, and the compact and dense aggregated area would lower the electrochemical reactivity, thus degrading the optical and EC properties. 

**Figure 6:**
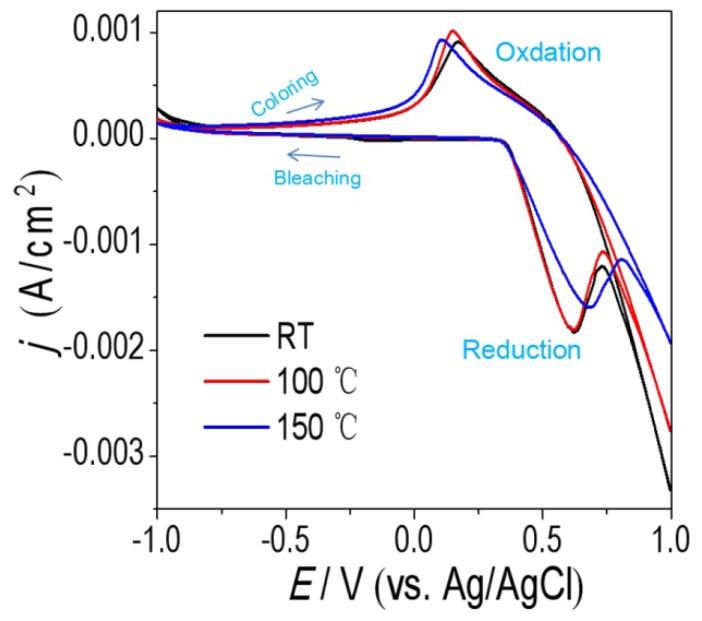
CVs of the NiO_x_ films annealed at RT, 100 °C and 150 °C in 1 M KOH electrolyte. The NiO_x_ films are spin-coated three times and the volume ratio in NiO_x_ inks is IPA:DI water = 1:3.

According to the above description, the NiO_x_ films annealed at RT, 100 °C and 150 °C have better Δ*T*. Therefore, their electrochemical properties were further characterized by cyclic voltammetry tests (CVs) at a scan rate of 100 mV/s. The results are shown in Figure 6. The shapes of the curves have typical oxidation and reduction peaks. The oxidation peaks correspond to coloring processes. The opposite reduction peaks correspond to bleaching processes. The NiO_x_ films annealed at 100 °C and 150 °C show much lower oxidation and reduction potentials compared to NiO_x_ film annealed at room temperature. Moreover, the NiO_x_ film annealed at 100 °C and 150 °C exhibits smaller potential separation between the oxidation peaks and the reduction peaks. It is well known that the peak potentials separation are used as a measure of reversibility [22]. It is reasonable that the NiO_x_ films annealed at 100 °C and 150 °C has better reaction reversibility. In addition, the cathodic and anodic peak current densities (*j*) of NiO_x_ films annealed at 100 °C and 150 °C are much higher than NiO_x_ film annealed at room temperature. It indicated that the NiO_x_ films annealed at 100 °C and 150 °C had higher electrochemical reaction activity. The amount of per unit charges (*Q*) in the insertion and extraction processes can be calculated [36]:

(9) $$Q=\frac{\int IdV}{v}$$

in which *I*, *v* and *V* are instantaneous current, and scan rate of CV curves and instantaneous potential, respectively. A parameter often used to characterize an EC material is the coloration efficiency (*CE*) [5], which is defined as the charge in Δ*OD*, per unit inserted charge density (*Q*_in_):

(10) $$CE\left(\lambda \right)=\frac{\Delta OD\left(\lambda \right)}{Q_{\mathrm{in}}}$$

the *CE* values of the NiO_x_ films annealed at room temperature, 100 °C and 150 °C at 550 nm wavelength are calculated to be 34.7 cm^2^/C, 36.7 cm^2^/C and 49.7 cm^2^/C, respectively.

**Figure 7:**
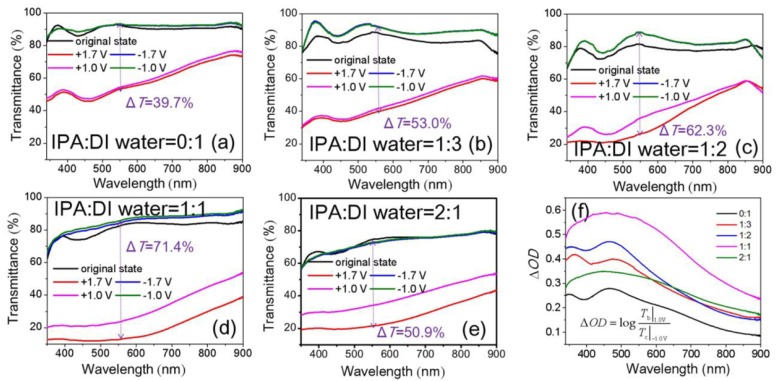
Optical transmittance spectra at as-deposited, colored and bleached states of NiO_x_ films as a function of the volume ratios of IPA:DI water in NiO*_x_* inks: (**a**) 0:1; (**b**) 1:3; (**c**) 1:2; (**d**) 1:1 and (**e**) 2:1 (The increases of voltage has little effect on the *T*_b_, so the 2 bleached curves overlap together) and (**f**) Δ*OD*. The NiO_x_ thin films are by spin-coating three times and after 150 °C annealing.

The optimum spin-coating times and annealing temperature of NiO_x_ films mentioned above are three times and 150 °C, respectively. On this basis, as IPA and DI water are used as dispersants for NiO_x_ NPs, we further focused on the effect of different volume ratios of IPA:DI water in NiO_x_ inks. Figure 7a–e show transmittance spectra of NiO_x_ films at colored and bleached states as a function of the volume ratios of IPA:DI water in NiO_x_ inks. With increasing IPA volume ratios, we observed a significant decrease of transmittance for colored states. However, there was only a small loss of bleached transmittance. The better Δ*T* value of 62.3% (IPA:DI water = 1:2) and 71.4% (IPA:DI water = 1:1) at 550 nm between the bleached states (−1.7 V) and the colored states (+1.7 V) were observed. The NiO_x_ films with the 1:2 and 1:1 ratios of IPA:DI water gave higher Δ*OD* compared to the others (Figure 7f).

**Figure 8:**
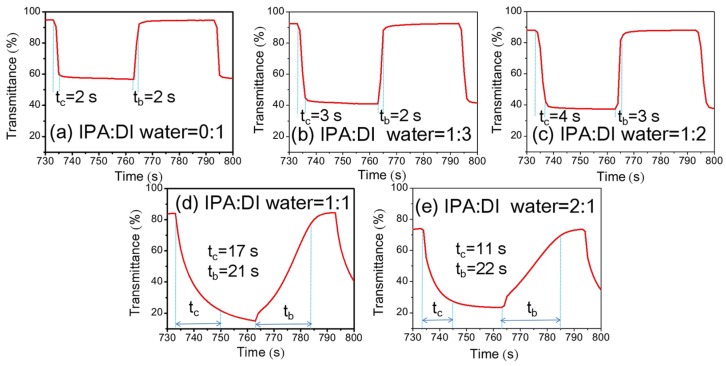
Switching curves of NiO_x_ films as a function of the volume ratios of IPA:DI water in NiO*_x_* inks: (**a**) 0:1; (**b**) 1:3; (**c**) 1:2; (**d**) 1:1 and (**e**) 2:1. The NiO_x_ films are by spin-coating three times and after 150 °C annealing.

Figure 8 shows the changes in transmittance at 550 nm of NiO_x_ films as a function of the volume ratios of IPA:DI water in NiO_x_ inks for applied square-wave-type voltages (±1.0 V, 60 s per cycle) at 550 nm. The volume ratios of IPA:DI water in NiO_x_ inks have a large effect on the switching time. When the volume ratios of IPA:DI water in NiO_x_ inks were 0:1, 1:3 and 1:2, fast switching speed was observed in Figure 8a–c. However, when the volume ratios of IPA:DI water in NiO_x_ inks were 1:1 and 2:1, we observed a significant extension of switching time to more than 10 s (Figure 8d,e).

**Figure 9:**
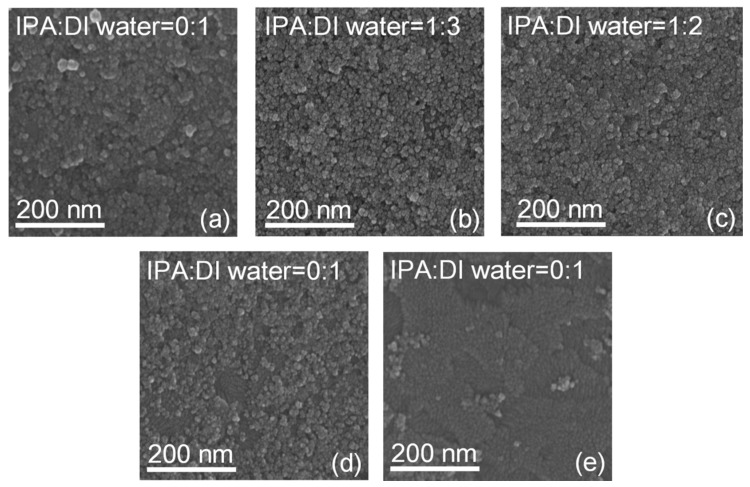
SEM images of NiO_x_ films in different volume ratios of IPA:DI water in NiO_x_ inks: (**a**) 0:1; (**b**) 1:3; (**c**) 1:2; (**d**) 1:1 and (**e**) 2:1. The NiO_x_ films are by three times spin-coating and after 150 °C annealing.

The effect of different volume ratios of IPA:DI water in NiO_x_ inks on Δ*T* and switching time of NiO_x_ films can be explained by SEM in Figure 9. When DI water was used as dispersant, NiO_x_ NPs could be evenly dispersed. However, due to the high surface tensions of DI water, cracks occurred more easily during annealing. When IPA was used as dispersant, its surface tension was small, which can overcome the shortcoming of DI water as dispersant. It could be seen that the NiO_x_ NPs were uniformly and distinctly dispersed on the substrate surfaces (Figure 9b,c). However, higher volume content of IPA in NiO_x_ inks typically cause fall-off of NiO_x_ NPs partially (Figure 9d,e), which reduces the relative surface area of NiO_x_ films and leads to the decrease of active reaction area.

**Figure 10:**
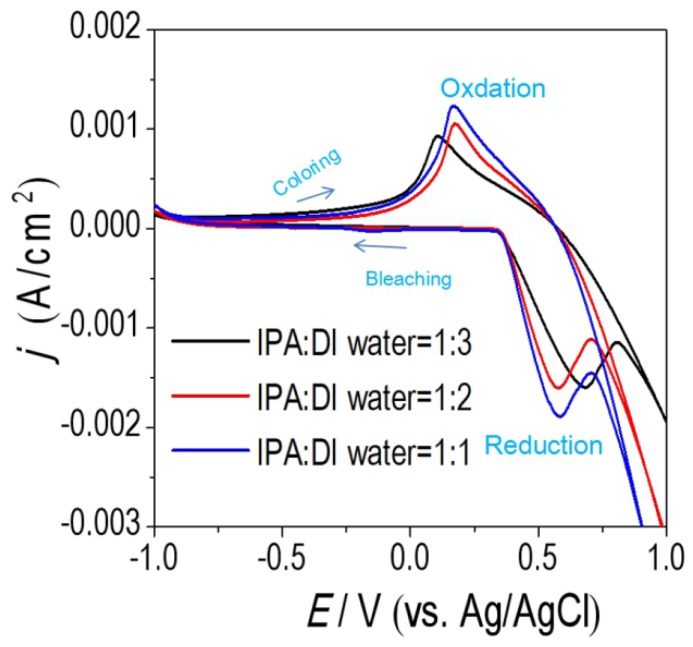
CVs of the NiO_x_ films in different volume ratios of IPA:DI water in NiO_x_ inks in 1 M KOH electrolyte. The NiO_x_ films are spin-coated three times and annealed to 150 °C.

The NiO_x_ films with 1:3, 1:2 and 1:1 volume ratios of IPA:DI water were further characterized by CVs at a scan rate of 100 mV/s. The results are shown in Figure 10. The NiO_x_ films with 1:2 and 1:1 volume ratios of IPA: DI water exhibit smaller potential separation between the oxidation peaks and the reduction peaks, so they have better reaction reversibility. Besides, the cathodic and anodic peaks *j* of the NiO_x_ films are much higher than NiO_x_ film with volume ratios of 1:3. It indicated that the NiO_x_ films with the volume ratios of 1:2 and 1:1 had higher electrochemical reaction activity. The *CE* values at 550 nm wavelength were calculated to be 49.7 cm^2^/C (IPA:DI water = 1:3), 42.8 cm^2^/C (IPA:DI water = 1:2) and 48.5 cm^2^/C (IPA:DI water = 1:1), respectively.

Table 1 summarizes the typical processing condition in references in the past 5 years. We can see that our method provides the lowest processing temperature, and superior EC properties than many works. We are aware that in many methods, formation of NiO are processed by decomposition of nickel salts (NiCl_2_, Ni(OAc)_2_, NiSO_4_, NiNO_3_, Ni(CH_3_COO)_2_) in dispersants when the NiO thin film was fabricated. In our method, we prepared NiO_x_ NPs first and fabricated the NiO_x_ films by the NiO_x_ NPs in dispersants. Dispersants with low boiling point were used, which ensured low-temperature deposition.

### 3.3. Cyclic Durability of the NiO_x_ Film with Optimized Parameters

**Figure 11:**
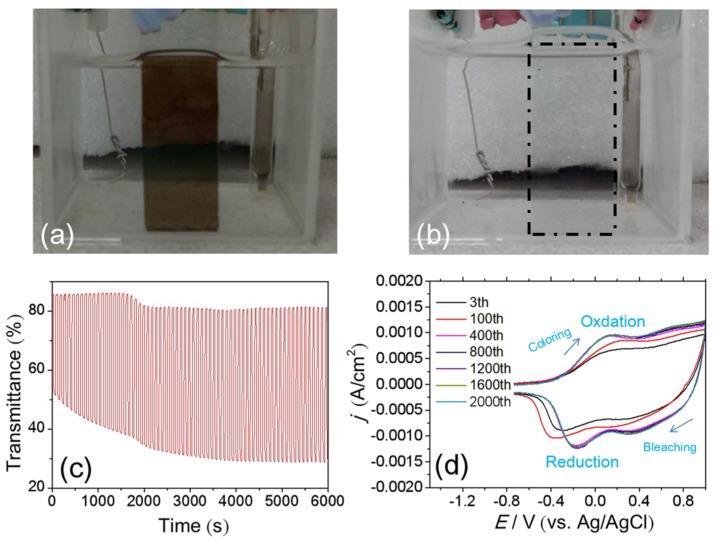
(**a**) Optical images of the colored and bleached states; (**b**) Optical images of the colored and bleached states; (**c**) cycles of optical switching for the optimized sample in 1 M KOH electrolyte and (**d**) CVs of the optimized NiO_x_ film in 0.5 M LiClO_4_-PC electrolyte.

Considering the above optimized parameters, the NiO_x_ film which is spin-coated three times and annealed at 150 °C with the 1:2 ratio in NiO_x_ inks of IPA:DI water gives better Δ*T*, higher *CE* and faster switching time. Therefore, we choose the optimized NiO_x_ film for further study. Figure 11a,b shows good contrast between the bleached state and the colored state, the NiO_x_ films exhibit reversible color change from dark brown (colored state) to transparent (bleached state). The optimized NiO_x_ electrode has been tested for applied square-wave-type voltages (±1.0 V, 90 s per cycle) up to 6000 s in 1 M KOH electrolyte. The spectral response at 550 nm has been recorded in Figure 11c. The Δ*T* of the NiO_x_ film exhibits a value of Δ*T* = 34.2% for the initial time. It increases gradually and reaches a maximum value of 52.7% in the steady period up to 6000 s. In addition, the cyclic durability limits the further advancement of NiO-based films as has been reported by many researchers, which showed that the degradations are depending on the applied electrolytes or fabrication parameters [13,37]. Therefore, we choose the optimized NiO_x_ electrode to further study its cyclic durability. Because NiO-based films are often used in ECDs containing Li^+^ electrolytes [38,39], we consider using 0.5 M LiClO_4_-PC electrolyte for cyclic voltammetry tests (CVs) in Figure 11d. It is usually recognized that NiO_x_ was subjected to the following electrochemical oxidation and reduction processes [13]:

(11) $${\mathrm{NiO}}_{x}+{\mathrm{yLi}}^{+}+{\mathrm{ye}}^{-}\to {\mathrm{Li}}_{y}{\mathrm{NiO}}_{x}$$

(12) $${\mathrm{Li}}_{y}{\mathrm{NiO}}_{x}↔{\mathrm{Li}}_{y-z}{\mathrm{NiO}}_{x}+{\mathrm{zLi}}^{+}+{\mathrm{ze}}^{-}$$

Figure 11d showed typical oxidation and reduction peaks. The peak potential shifted in the first few cycles, when the migration paths for ions were established and became stable. This process involved the slight change of thin film resistance, which caused slight change of voltage on the film, and thus the shifting of reduction and oxidation peaks. The peaks became immobile after 400 cycles. The separation between cathodic and anodic peaks of *j* in the steady periods was smaller than that of the initial periods. It indicated that the NiO_x_ films had higher electrochemical reaction activity in the steady periods. It showed that the optimized NiO_x_ film did not obviously degraded until 2000 cycles, which proved good stability of the optimized NiO_x_ film in Li^+^-based electrolytes.

## 4. Conclusions

In summary, NiO_x_ NPs fabricated by chemical precipitation method were developed to prepare IPA-DI water-NiO_x_ suspensions for application of spin-coated NiO_x_ films. The optical properties of EC NiO_x_ films as functions of the number of spin-coating layers, annealing temperature, and the volume ratios of IPA to DI water in IPA-DI water-NiO_x_ suspensions were systematically investigated. Large transmittance of 62.3% at 550 nm, high coloration efficiency (42.8 cm^2^/C), rapid switching time (coloring time is 4 s, bleaching time is 3 s) and good stability were achieved in the optimized NiO_x_ film, which is characterized by spin-coating three times and 150 °C annealing in air with the 1:2 volume ratio of IPA:DI water. We showed that the NiO_x_ NPs were uniformly and distinctly dispersed in the optimized NiO_x_ film, which facilitated the best ion migration. CVs of the optimized NiO_x_ film in LiClO_4_-PC electrolyte prove to have good cyclic durability without obvious degradation. The fabrication technique used low-cost and non-toxic precursors with low fabrication temperature, which facilitates further development of fabricating electrochromic devices at low temperatures.

## Figures and Tables

**Table 1:** NiO-based films reported earlier by chemical solution methods in KOH electrolyte.

No.	Methods	Films	Annealing Condition	Δ*T* (%)	*CE* (cm^2^/C)	*T*_c_/*T*_b_ (s)	Ref.	Year
1	Dip-coating	NiO film	500 °C	51	40	7/5	[15]	2017
2	Dip-coating	NiO film	350 °C	50.7	71.4	-	[16]	2017
3	Inkjet printing	NiO film	200 °C	64.2	136.7	9/6	[12]	2016
4	Hydrothermal	NiO film	300 °C	35.8	49.8	1.3/3.2	[20]	2015
5	Hydrothermal	NiO film	400 °C/Ar	40	63.2	2.7/1.8	[21]	2015
6	Hydrothermal	NiO film	300 °C/Ar	77	49	3/4	[22]	2013
7	Spin-coating	Al-doped NiO film	400 °C	58.4	54.2	4.2/1.8	[18]	2016
8	CBD	Co-doped NiO film	300 °C	88.3	47.7	5.4/3.4	[23]	2014
9	Dip-coating	NiO/GO film	350 °C	40.7	12.85	4.3/3.9	[27]	2017
10	Spin-coating	Ni/NiO/rGO film	350 °C/N_2_ and air	51.6	48.15	4.2/2.4	[19]	2017
11	CBD	TiO_2_/NiO film	300 °C/Ar	83	60.6	6.8/14.8	[24]	2014
12	Spin-coating	NiO_x_ film	150 °C/air	62.3	42.8	4/3	This work	
